# Global trends and hotspots of intra-articular hyaluronic acid therapy for knee osteoarthritis: A bibliometric study

**DOI:** 10.1097/MD.0000000000050660

**Published:** 2026-09-11

**Authors:** Zhijun Yang, Penghui Li, Yu Xu, Feifei Shen, Jie Li, Youkang Dong

**Affiliations:** a First Clinical Medical College, Yunnan University of Chinese Medicine, Kunming, Yunnan, China; b Second Clinical Medical College, Yunnan University of Chinese Medicine, Kunming, Yunnan, China; c Department of Tuina, Qujing Hospital of Traditional Chinese Medicine, Qujing, Yunnan, China; d College of Ethnic Medicine, Yunnan University of Chinese Medicine, Kunming, Yunnan, China; e Department of Tuina, The First Affiliated Hospital of Yunnan University of Chinese Medicine, Kunming, Yunnan, China.

**Keywords:** bibliometric analysis, intra-articular hyaluronic acid, knee osteoarthritis, knowledge-domain mapping, mesenchymal stem cells, regenerative medicine

## Abstract

**Background:**

Knee osteoarthritis (KOA) imposes a substantial global socioeconomic burden. Intra-articular hyaluronic acid (IAHA) injections are widely used as part of conservative management strategies for KOA; however, their clinical application and underlying mechanisms remain topics of ongoing research and debate.

**Methods:**

A bibliometric analysis of publications related to IAHA for KOA from 2015 to 2025 was conducted using the Web of Science Core Collection, with data retrieved on November 15, 2025. CiteSpace, VOSviewer, and the bibliometrix R package were used to analyze publication trends, collaborative networks, keyword co-occurrence, and emerging research themes.

**Results:**

A total of 980 publications were included. The annual number of publications showed an overall increasing trend over the past decade, with China and the United States contributing the highest publication output. Keyword co-occurrence and burst analyses indicated growing research interest in regenerative medicine-related topics, particularly mesenchymal stem cells, platelet-rich plasma, and combination-based therapeutic approaches. Collaborative network analyses also demonstrated increasing international research activity, although cross-regional cooperation remained relatively limited.

**Conclusion:**

Research on IAHA for KOA has expanded steadily over the past decade, with increasing attention directed toward regenerative and combination-based therapeutic strategies. Current bibliometric findings suggest evolving academic interest in mechanistic exploration and multimodal treatment approaches, while highlighting the need for further high-quality collaborative studies in this field.

## 1. Introduction

Knee osteoarthritis (KOA) is a common form of chronic degenerative joint disease and is a significant contributor to worldwide disability, posing a continuous burden to healthcare resources.^[1,2]^ Often misunderstood as a form of “cartilage wear and tear,” KOA is better understood as a disease process affecting the entire joint. The process of disease progression is marked by a loss of joint cartilage, resulting from damage to the extracellular matrix and aberrant remodeling of subchondral bone tissue.^[3]^ These tissue changes are directly associated with pain, episodic synovial inflammation, and functional deterioration. As disease progression continues, joint instability and decreased mobility increasingly limit functional independence and quality of life.

In China, for instance, epidemiological surveys show that KOA prevalence stands at 18% of the population, with a consistently higher prevalence of KOA reported among women than men. With the country undergoing a rapid transition to an aging population, KOA is no longer just considered a chronic condition to be managed but has been positioned as a rising public health concern.^[4]^ In this context, it becomes important to recognize the changing therapeutic landscape and identify what has the capacity to bring relief to patients with KOA and reduce the overall public health and economic burden. Intra-articular hyaluronic acid (IAHA) injection has been identified as one of the most commonly used treatments for KOA. Although the body of evidence has been expanding over the years, there are still uncertainties with regard to the duration of benefit, injection regimens, and mechanisms of action. While existing syntheses have focused on summarizing the body of evidence with regard to the pooled effects of IAHA injection for KOA, the field-level perspective has not been synthesized under one roof. A bibliometric view can therefore complement conventional meta-analyses by showing how the evidence base is evolving and where attention is currently moving.

As publication volume grows, narrative reviews alone may overlook subtle changes in emphasis or the structure of collaboration. Bibliometric and science-mapping approaches offer a quantitative, reproducible way to track trends and to visualize intellectual linkages through citation networks and keyword co-occurrence. Although these tools do not replace systematic review, they can be used to gain insight into influential founding sources and rapidly evolving topics, including fleeting “bursts” of scholarly investigation.

Accordingly, our investigation utilized a multi-tool bibliometric approach to analyze the global IAHA literature on KOA from 2015 to 2025, utilizing CiteSpace,^[5]^ VOSviewer,^[6]^ and the bibliometrix R package.^[7]^ The goals of this overview were to describe publication trends and geographic locations, determine the major thematic clusters that organize the field, and detect emerging topics through keyword burst analysis, providing a framework to guide clinicians and researchers in understanding the current state of the art and future direction of hyaluronic acid-related therapies. The period from 2015 to 2025 was selected to capture the most recent decade of IAHA-related research, during which increasing attention has been paid to comparative injection therapies, regenerative medicine, and combination-based approaches. Although earlier studies established the historical basis of IAHA use, the selected interval allowed us to focus on contemporary research trends and emerging topics relevant to current clinical and academic discussions.

## 2. Materials and methods

### 2.1. Data source and search strategy

In order to ensure the collection of high-quality, comprehensive bibliographic information, the data were collected from the Web of Science Core Collection (WoSCC), considered the gold standard for comprehensive bibliometric study. A well-designed search strategy was executed on November 15, 2025, using the advanced search feature of the database to maximize the effectiveness of the search. The final Boolean query was designed as follows: (TS = ((“hyaluronic acid” OR “sodium hyaluronate” OR “viscosupplementation”) AND (“injection” OR “intra-articular injection”) AND (“knee osteoarthritis” OR “knee OA” OR “osteoarthritis of the knee”))).

**Figure 1.**
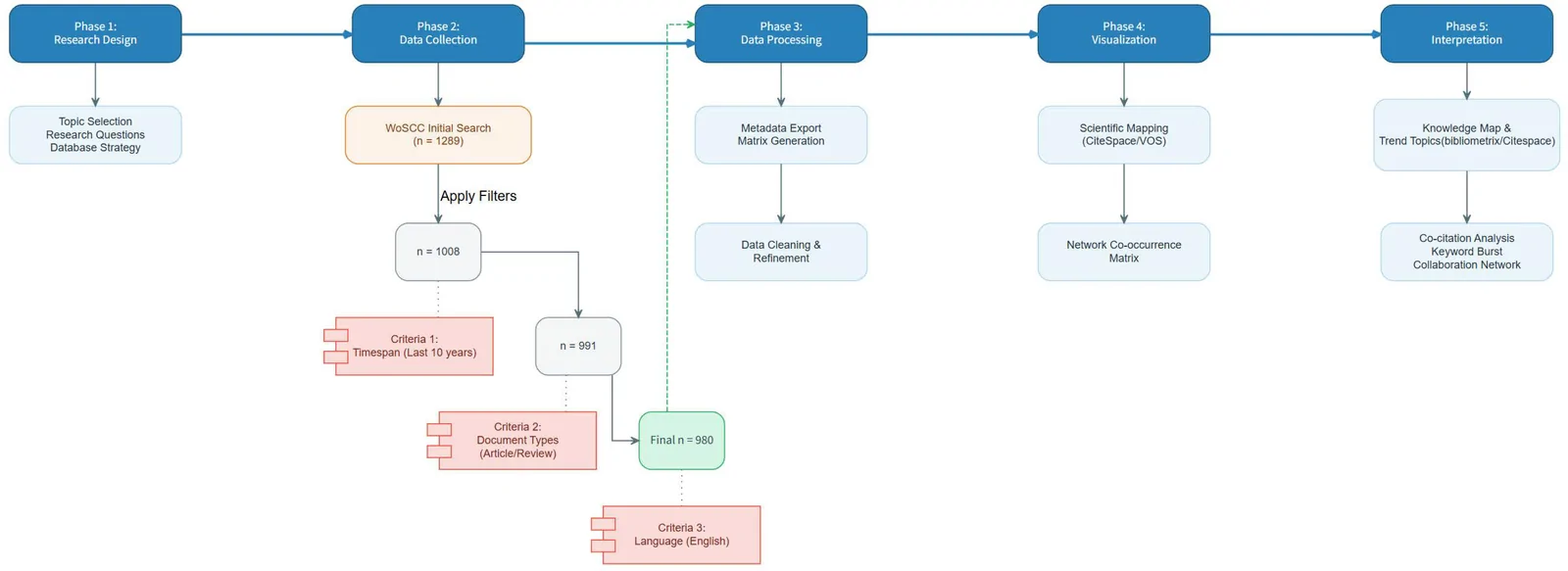
Integrated methodological framework illustrating the systematic literature retrieval, eligibility screening, and multidimensional bibliometric visualization process. WoSCC = Web of Science Core Collection.

The span of time was set from January 1, 2015, to November 15, 2025, to capture recent developments over approximately the past decade. We included only “Article” and “Review” document types and excluded items such as editorials, letters, and meeting abstracts to reduce noise from non–peer-reviewed or preliminary content. To ensure consistency in text processing and network visualization, we restricted the dataset to English-language publications. The study selection procedure and the subsequent analysis pipeline are summarized in Figure 1.

### 2.2. Data retrieval and preprocessing

All records were retrieved from WoSCC with complete bibliographic metadata (authors, title, journal, year, country/region, institutions, and keywords). Data were exported as plain-text (.txt) files including “Full Record and Cited References” for subsequent network analyses. Journal impact factors (IFs) were referenced to the 2024 Journal Citation Reports (JCR).

Country/region and institutional names were standardized prior to analysis to improve consistency in collaboration mapping. Keyword normalization was manually performed before network construction by merging synonyms, abbreviations, spelling variants, and hyphenated/non-hyphenated forms into unified terms. For example, “intraarticular injection” and “intra-articular injection” were unified as “intra-articular injection,” while “PRP” and “platelet-rich plasma” were merged as “platelet-rich plasma.” Similarly, “mesenchymal stem-cells” was standardized as “mesenchymal stem cells,” and “metaanalysis” was unified as “meta-analysis.” The complete keyword normalization dictionary used in this study is provided in Table S1, Supplemental Digital Content 1. During the final data-cleaning stage, synonymous keyword variants were standardized before network construction. Specifically, “articular-cartilage” was merged into the unified term “cartilage.” The thesaurus-based normalization procedure was applied in VOSviewer before the keyword co-occurrence analysis.

Microsoft Excel supported the initial curation, whereas CiteSpace and VOSviewer were used for visualization; bibliometrix was used to compute descriptive indicators.

### 2.3. Visual analysis and mathematical modeling

#### 2.3.1. Temporal trajectory analysis

The annual number of publications and the trends over time were created using Microsoft Excel 2024 and the ggplot2 library version 4.0.0 (Posit Software, PBC) for R. The plots were used to illustrate the changes in publications over the years and to identify the years when the growth in publications accelerated or leveled off.

#### 2.3.2. Collaborative architecture and co-occurrence mapping

We utilized the VOSviewer application (version 1.6.20; Centre for Science and Technology Studies, Leiden University) to visualize the collaboration patterns in the subject area. Instead of providing the list of contributors, we created co-authorship networks at the country/region, institution, and author levels, as well as keyword co-occurrence networks, to understand the thematic content of the subject area. We examined the network, overlay, and density views of the network visualization, where we could track the evolution of the research hotspots over time, as well as the concentration of topics. In the network visualization, the size of the nodes represents the citation frequency or keyword frequency, while the thickness of the links represents the strength of the relationship, and the color represents the cluster of the topics.

#### 2.3.3. Dynamic knowledge mapping via CiteSpace

To gain further insights into the structure and dynamics of the knowledge domain, science mapping analyses were performed using CiteSpace (version 6.4.R1; Chaomei Chen, Drexel University). The analyses included author collaboration network visualization, dual-map overlays of journal distribution, keyword cluster timeline visualization, and document co-citation network analysis. Keyword burst detection was additionally applied to identify emerging research topics and influential references.

The time span from January 2015 to November 2025 was analyzed using 1-year time slices. Network construction was performed using a scaling factor of *k* = 25, local reference frequency = 2.5, and look-back years set to 8 or 5 years depending on the analysis. The elasticity factor (*e*) was set to 1.0. To simplify the network structure and improve interpretability, minimum spanning tree pruning was applied during network visualization.

#### 2.3.4. Thematic evolution and global distribution

To further examine thematic evolution, we used bibliometrix (v5.1.1; Massimo Aria and Corrado Cuccurullo, University of Naples Federico II) in R (v4.5.1; R Foundation for Statistical Computing) to generate visualizations of global distribution and collaboration patterns. We also employed the Biblioshiny interface, using the “Trend Topics” module to track changes in frequently occurring themes over time and to visualize the emergence of research hotspots.

## 3. Results

### 3.1. Analysis of annual publication volume

**Figure 2.**
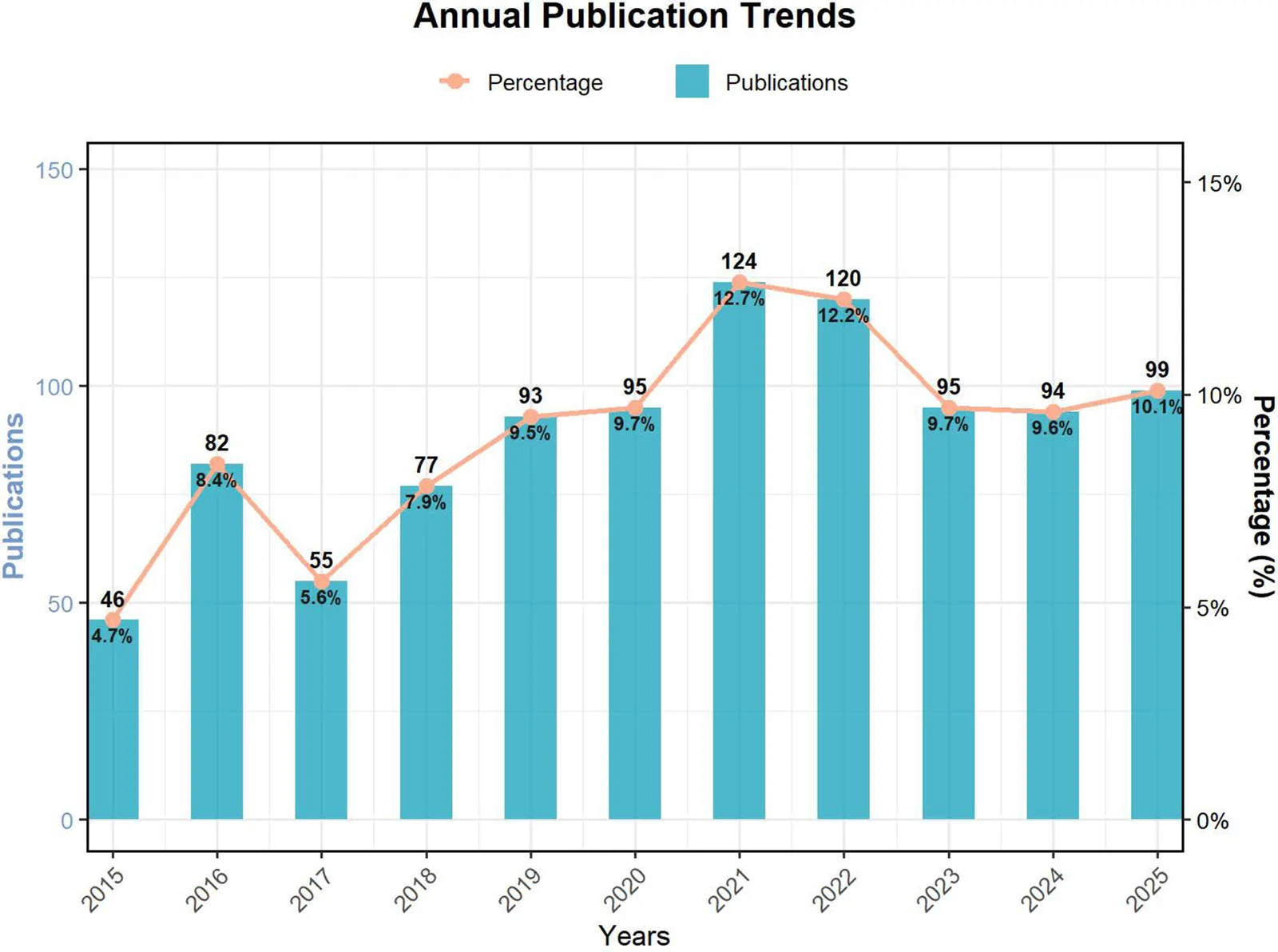
Global publication volume and evolutionary trends of research on HA injections for KOA (2015–2025). HA = hyaluronic acid, KOA = knee osteoarthritis.

Across 980 publications, the annual output indicates a rising and then stabilizing pattern (Fig. 2). After an initial phase with ~64 papers/yr in 2015 to 2016, publication volume increased markedly from 2018 and reached its highest level in 2021. In subsequent years, output has remained relatively stable (~102 papers/yr), implying that the field has moved from rapid expansion toward a more steady production phase.

### 3.2. Analysis by countries and institutions

Table 1 shows how many publications are made in each country. China contributed the largest number of publications (n = 249, 25.41%), followed by the United States (n = 200, 20.41%), Italy (n = 90, 9.18%), and South Korea (n = 44, 4.49%). China and the United States combined published 45.82% of all articles. This suggests that a lot of research is being done in these 2 countries.

**Table 1:** Ranking of the top 10 most productive countries and regions in research on IAHA for knee osteoarthritis (2015–2025).

Rank	Country/region	Publication count (%)
1	China	249 (25.41%)
2	The United States	200 (20.41%)
3	Italy	90 (9.18%)
4	South Korea	44 (4.49%)
5	France	39 (3.98%)
6	Turkey	32 (3.27%)
7	Spain	27 (2.76%)
8	Japan	26 (2.65%)
9	Iran	25 (2.55%)
10	Australia	23 (2.35%)

IAHA = intra-articular hyaluronic acid.

**Figure 3.**
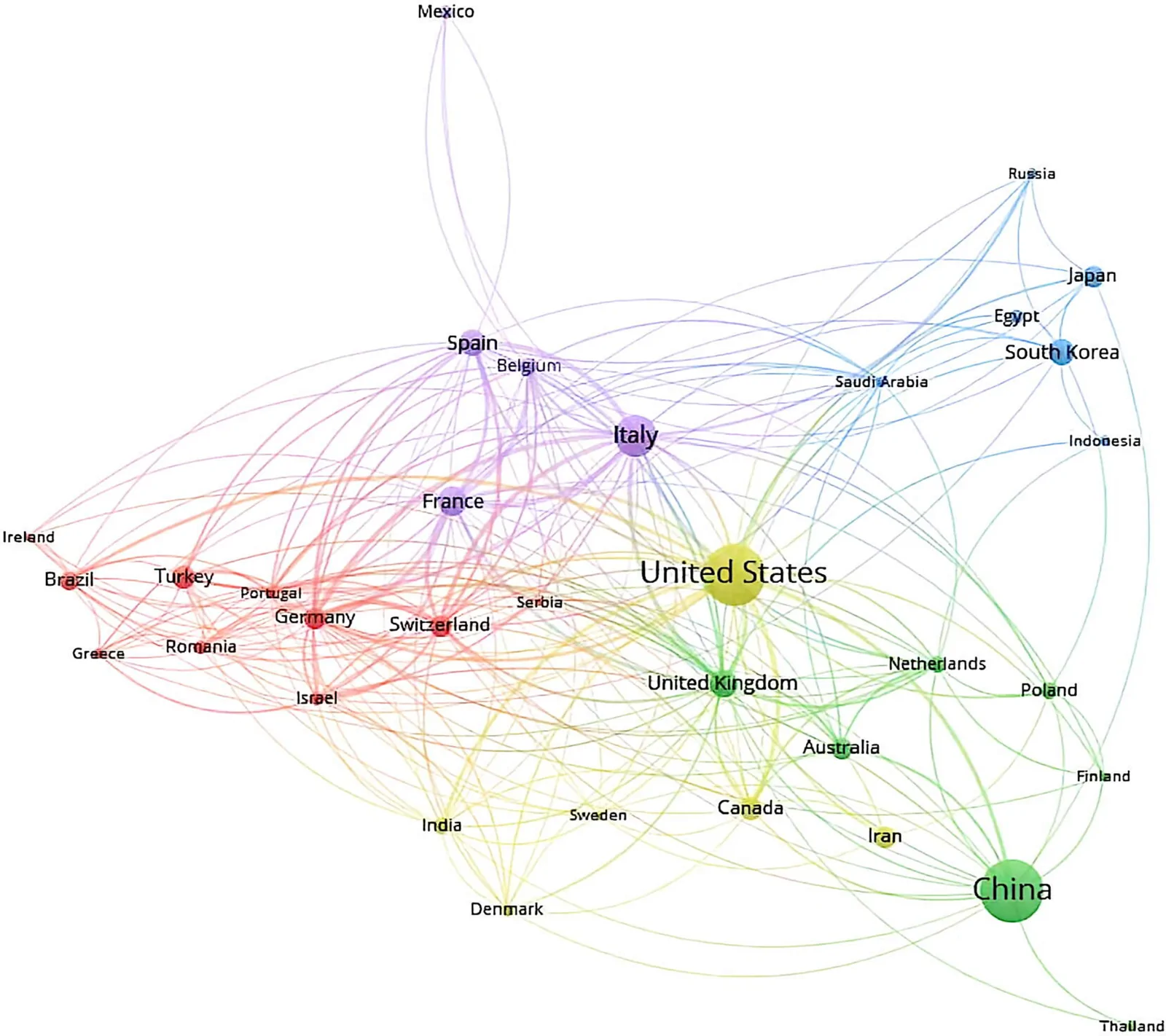
Global collaborative network map of countries and regions based on co-authorship analysis.

**Figure 4.**
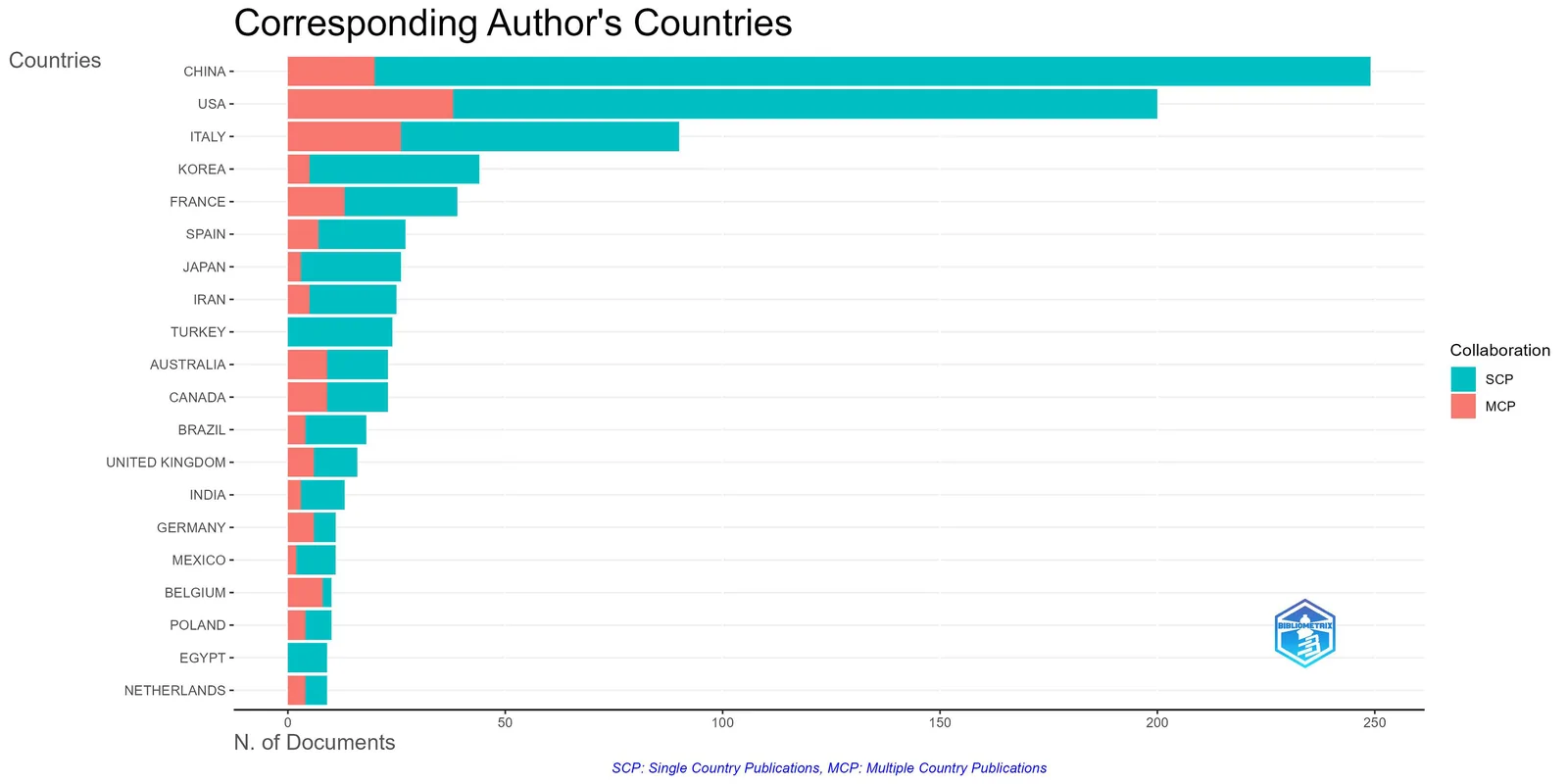
Geopolitical distribution of corresponding authors among the top 20 most productive nations. MCP denotes international collaborative research output, while SCP signifies intranational scholarly production. MCP = multiple-country publication, SCP = single-country publication.

We created an international co-authorship network in VOSviewer (Fig. 3) to examine how people work together. The map indicates many well-formed clusters around key research hubs, but there appear to be few cross-national links, which suggests that international collaboration is still restricted in scope. This pattern is consistent with the corresponding-author distribution produced in bibliometrix (Fig. 4). In particular, output is dominated by single-country publications, whereas multiple-country publications – a proxy for international co-production – represent a smaller share of the total. Taken together, these findings suggest that the field has strong national-level productivity but has not yet developed a highly integrated global collaboration structure.

Institutional productivity analysis showed that Rush University (USA) was the most prolific organization, contributing 25 publications (2.55%). To characterize collaboration among institutions, we constructed an institutional co-authorship network in VOSviewer and set a minimum threshold of 5 publications; 93 institutions met this criterion and were included in the analysis to highlight the main collaboration structure of the field.

**Figure 5.**
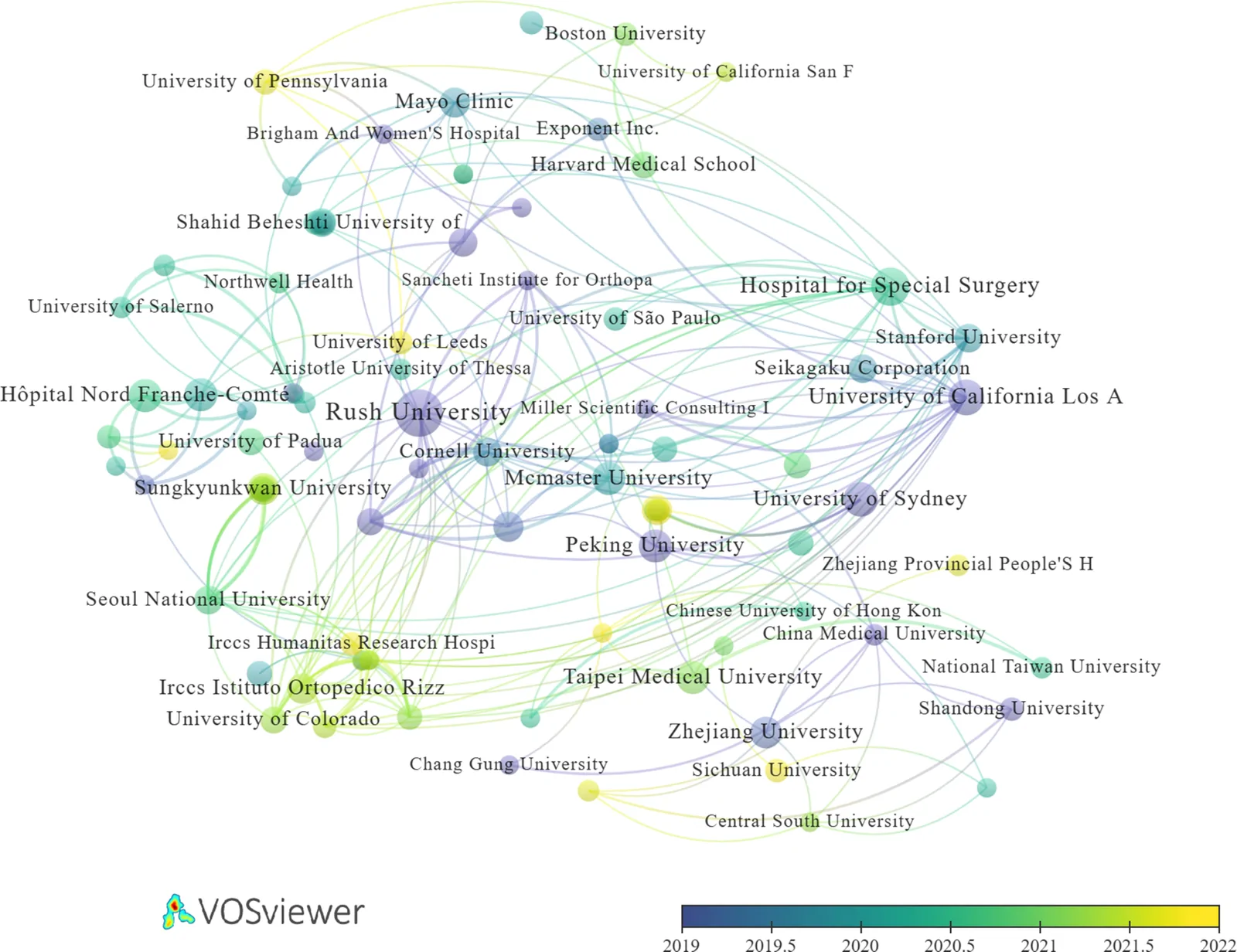
Institutional co-authorship network map of publications in the field of IAHA for knee osteoarthritis. IAHA = intra-articular hyaluronic acid.

As shown in Figure 5, the network forms several distinct clusters, with a core group of frequently collaborating institutions. Some of the most important nodes include Rush University, Cornell University, the University of Washington, and the University of Toronto. These institutions occupy central positions within the network and show relatively dense collaborative connections. This makes it likely that most of the collaborative work is done at a small number of high-output academic and medical facilities, mostly in North America and Europe. This clustering pattern suggests that there is already a way for different centers to work together, which could make it easier for researchers to work together and share IAHA-related clinical and methodological practices.

### 3.3. Distribution profiles and co-citation architecture of scholarly journals

#### 3.3.1. Distribution characteristics of source journals

The 980 articles were published in 326 journals that are indexed by WoSCC. The top 15 most productive journals (Table 2) were primarily oriented toward orthopedics, clinical medicine, surgery, and sports medicine, indicating that IAHA research in KOA remains anchored in clinically driven disciplines. In terms of journal metrics, most of these journals were categorized within JCR/CAS Q1 to Q3 classifications in their respective research areas, with IFs generally below 10.

**Table 2:** The top 15 most productive journals publishing research on IAHA for knee osteoarthritis (2015–2025).

Rank	Journal	Count (N)	IF (Q)
1	BMC Musculoskeletal Disorders	51 (5.20%)	2.4 (Q2)
2	Journal of Clinical Medicine	30 (3.06%)	2.9 (Q1)
3	Arthroscopy: The Journal of Arthroscopic and Related Surgery	27 (2.76%)	5.4 (Q1)
4	American Journal of Sports Medicine	25 (2.55%)	4.5 (Q1)
5	Cartilage	25 (2.55%)	2.7 (Q2)
6	Journal of Orthopaedic Surgery and Research	23 (2.35%)	2.8 (Q1)
7	Medicine	23 (2.35%)	1.4 (Q2)
8	Orthopaedic Journal of Sports Medicine	22 (2.24%)	2.5 (Q2)
9	International Journal of Molecular Sciences	17 (1.73%)	4.9 (Q1)
10	Knee Surgery, Sports Traumatology, Arthroscopy	16 (1.63%)	5.0 (Q1)
11	Journal of Pain Research	14 (1.43%)	2.5 (Q2)
12	Clinical Rheumatology	13 (1.33%)	2.8 (Q2)
13	PLoS One	13 (1.33%)	2.6 (Q2)
14	Osteoarthritis and Cartilage	12 (1.22%)	9.0 (Q1)
15	Journal of Arthroplasty	11 (1.12%)	3.8 (Q1)

IAHA = intra-articular hyaluronic acid, IF = impact factor, Q = quartile.

This journal distribution likely reflects the translational character of the field: much of the literature is focused on applied clinical questions (e.g., symptom control, comparative regimens, and practice-oriented outcomes), which are commonly published in specialty journals rather than broad, high-impact general medical venues. At the same time, journal rank alone does not determine scientific value; it may also mirror heterogeneity in trial design, variability in patient selection, and differences in outcome definitions that make findings harder to consolidate into practice-changing evidence. Moving forward, higher-quality evidence will probably depend on well-designed, adequately powered multicenter trials, clearer reporting standards (including protocol registration and harmonized outcomes), and stronger cross-institutional collaboration – elements that would increase both credibility and the likelihood of publication in higher-impact journals.

#### 3.3.2. Analysis of journal citation frequency and scholarly impact

Journal co-citation analysis (Table 3) highlights a small set of highly influential venues, with 5 journals exceeding 1000 total co-citations. Osteoarthritis and Cartilage ranked first (2972 co-citations), followed by the American Journal of Sports Medicine (2030), Arthroscopy (1410), Annals of the Rheumatic Diseases (1296), and Knee Surgery, Sports Traumatology, Arthroscopy (1076). These frequently co-cited sources are likely to be important knowledge bases, repeatedly used for framing IAHA-related KOA research.

**Table 3:** Ranking of the top 15 most influential co-cited journals in research concerning IAHA for knee osteoarthritis (2015–2025).

Rank	Journal	Total citations	IF (Q)
1	Osteoarthritis and Cartilage	2972	9.0 (Q1)
2	American Journal of Sports Medicine	2030	4.5 (Q1)
3	Arthroscopy: The Journal of Arthroscopic and Related Surgery	1410	5.4 (Q1)
4	Annals of the Rheumatic Diseases	1296	20.6 (Q1)
5	Knee Surgery, Sports Traumatology, Arthroscopy	1076	5.0 (Q1)
6	BMC Musculoskeletal Disorders	929	2.4 (Q2)
7	Journal of Bone and Joint Surgery-American Volume	819	4.4 (Q1)
8	Arthritis & Rheumatology	725	10.9 (Q1)
9	Journal of Rheumatology	672	3.4 (Q2)
10	Seminars in Arthritis and Rheumatism	606	4.4 (Q1)
11	Arthritis Research & Therapy	596	4.6 (Q1)
12	International Journal of Molecular Sciences	483	4.9 (Q1)
13	Clinical Orthopaedics and Related Research	461	4.5 (Q1)
14	PLoS One	443	2.6 (Q2)
15	Cartilage	442	2.7 (Q2)

IAHA = intra-articular hyaluronic acid, IF = impact factor, Q = quartile.

In terms of journal-level metrics, Annals of the Rheumatic Diseases had the highest IF among the listed venues (IF = 20.6), followed by Arthritis & Rheumatology (IF = 10.9). The co-citation profile and journal metrics show that the discipline draws many of its ideas from both rheumatology and orthopedics/sports medicine due to overlapping disease biology, clinical management, and procedure-oriented research.

**Figure 6.**
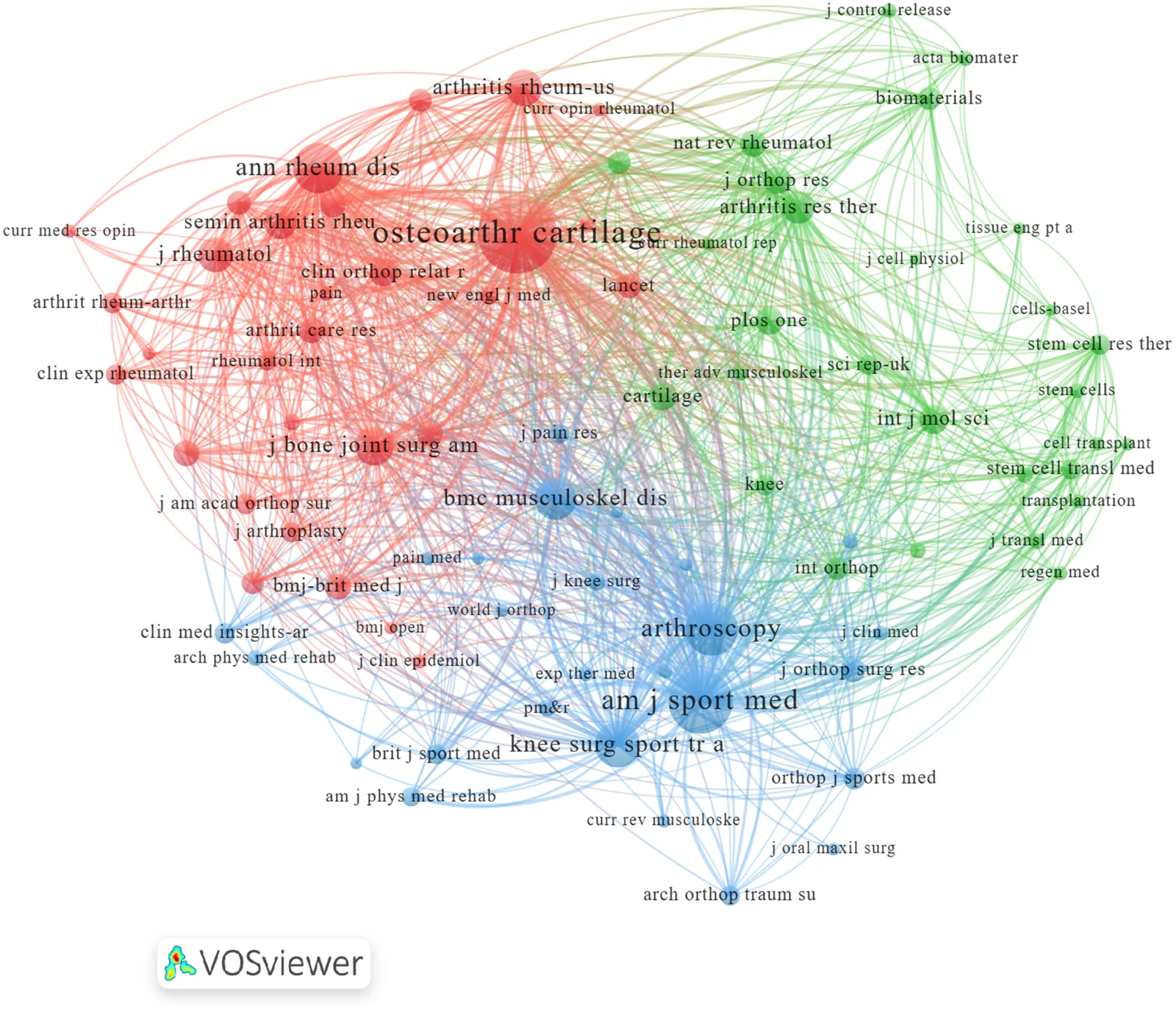
Journal co-citation network map illustrating the intellectual foundations of research on IAHA for knee osteoarthritis. IAHA = intra-articular hyaluronic acid.

Using a minimum co-citation threshold of 90, we constructed a journal co-citation network in VOSviewer (Fig. 6). The network places Osteoarthritis and Cartilage, Arthritis & Rheumatology, and The Journal of Bone and Joint Surgery-American Volume in central positions, indicating their strong connectivity to the broader literature. The presence of general medical and evidence-synthesis outlets (e.g., the Cochrane Database of Systematic Reviews, The New England Journal of Medicine, and The Lancet) suggests that KOA and its interventions are discussed beyond specialty journals, particularly in the context of evidence appraisal and clinical relevance; however, co-citation alone should be interpreted as an index of intellectual linkage rather than a direct measure of public health priority.

The existence of a cluster encompassing Biomaterials and Stem Cells indicates that IAHA research is increasingly integrating with the biomaterials and regenerative medicine literature. This tendency aligns with a move away from standard viscosupplementation and toward approaches that focus on biologics and repair. The journal co-citation network shows how mechanistic and materials-oriented work in the field is linked to clinical therapies, pain and function results, and higher-level evidence synthesis.

#### 3.3.3. Dual-map overlay visualization: tracing the intellectual trajectories of knowledge flow

The journal dual-map overlay provides a macroscopic view of cross-disciplinary citation patterns by linking citing journal groups (left) to cited journal groups (right) through colored trajectories.^[8]^ In this map, the left-side clusters indicate where the included IAHA–KOA studies are published, whereas the right-side clusters reflect the disciplinary areas most frequently referenced by those studies. The connecting paths visualize major knowledge flows between these domains and offer an intuitive summary of how evidence is built across fields.

**Figure 7.**
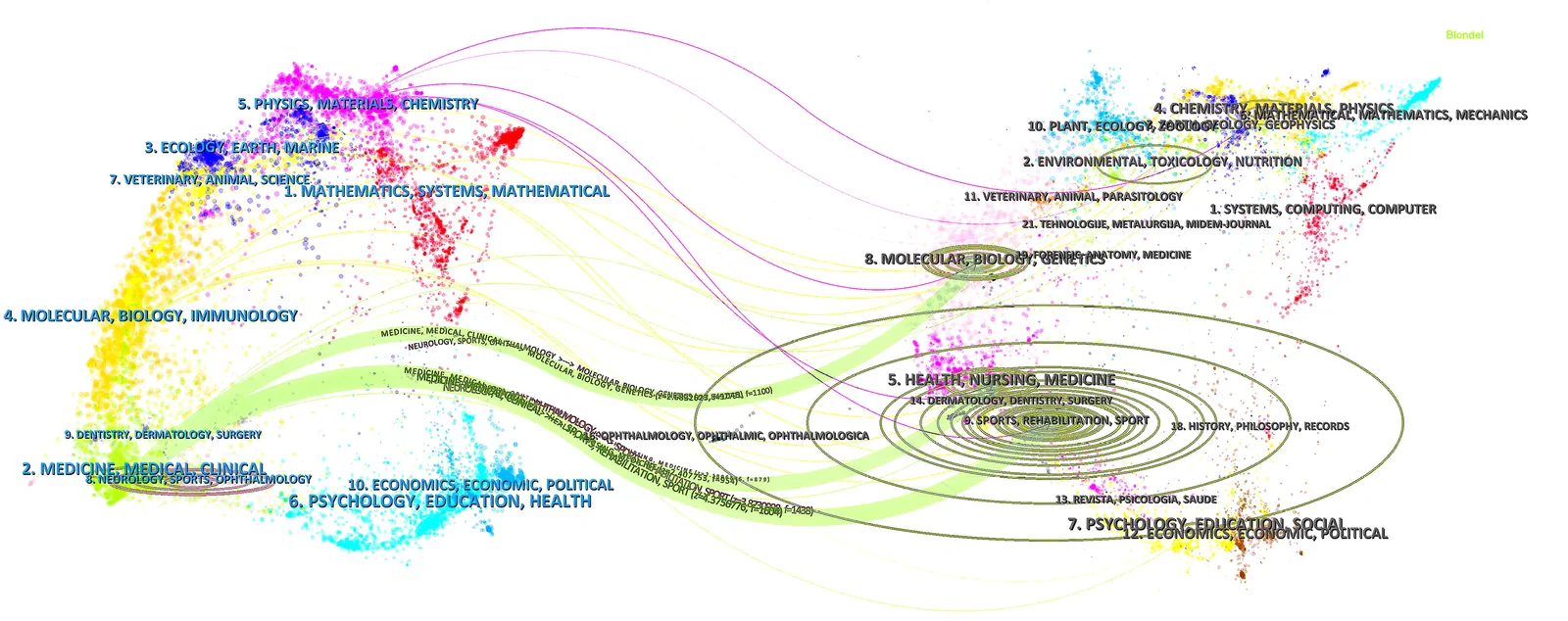
Dual-map overlay of journals associated with IAHA for knee osteoarthritis. IAHA = intra-articular hyaluronic acid.

As shown in Figure 7, the citing journals are primarily concentrated in Medicine/Medical/Clinical-related clusters (including clinical immunology–adjacent areas), suggesting that the recent IAHA literature is largely disseminated through clinically oriented outlets. This pattern is consistent with a largely patient-oriented research agenda, with an emphasis on symptom relief, functional improvement, and treatment optimization.

The journals cited in these groups represent a more general range of disciplines, from molecular and cellular biology to chemistry/materials science, biomechanics/mechanics, and mathematics/computation. This distribution suggests that the knowledge base of IAHA research is quite broad; basic science can often be used to support the biological plausibility of human studies, whereas mathematics and the quantitative fields can provide tools to support modeling, measurement, and methodological development. Interestingly, the overlap does not suggest a traditional “bench to bedside” model; rather, it suggests an ongoing interplay between human problem-solving and mechanistic/methodological investigation. From this perspective, the cross-citation structure supports the growing interest in biomaterials and regenerative approaches, where mechanistic understanding and more robust quantification are becoming increasingly important.

### 3.4. Analysis of prolific authors and co-authorship networks

**Figure 8.**
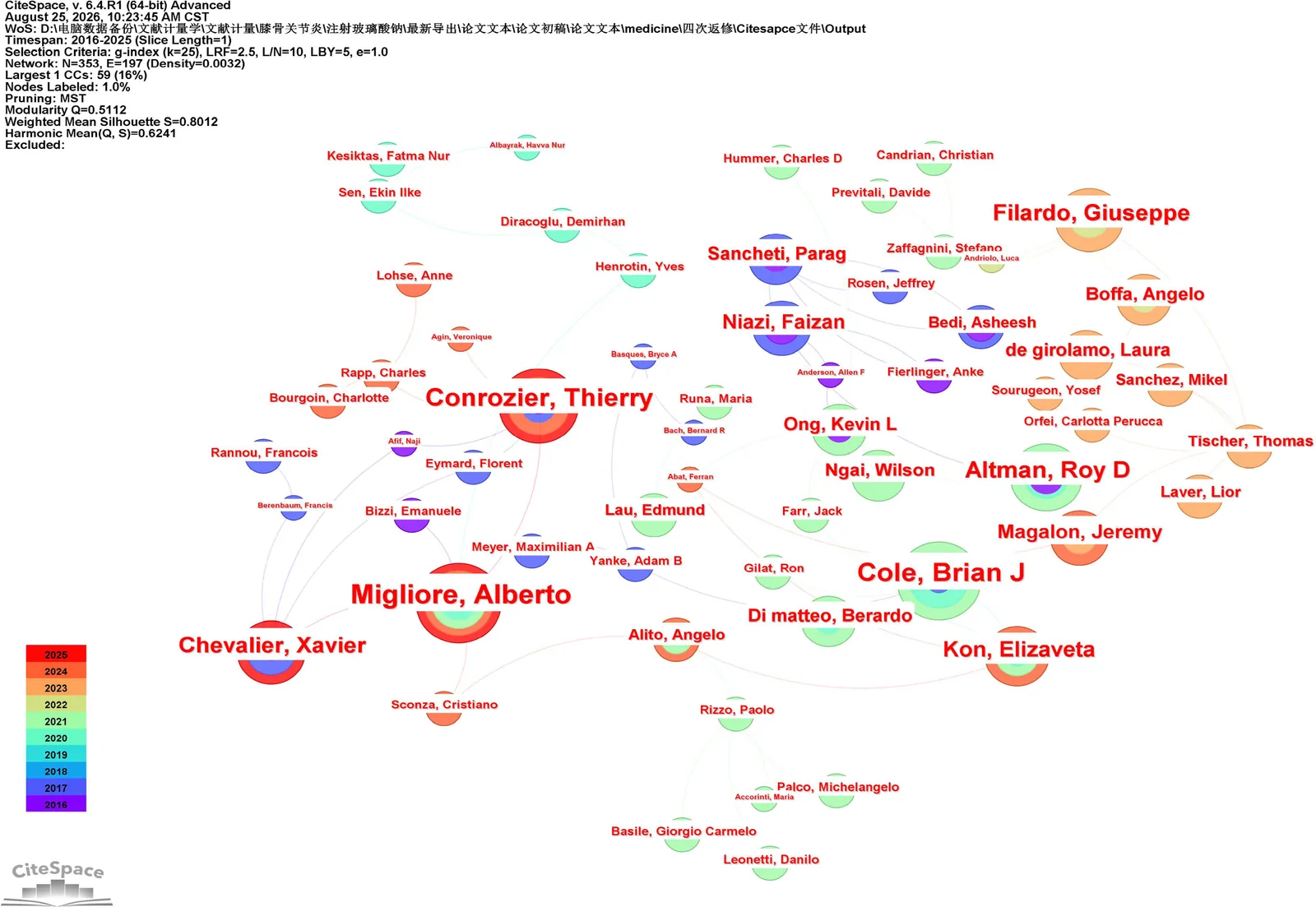
Author collaboration network map illustrating the global research community in the field of IAHA for knee osteoarthritis. IAHA = intra-articular hyaluronic acid.

The authorial analysis (Table 4) shows that the most prolific author is Alberto Migliore with 17 publications. The coauthor network (Fig. 8) shows a discernible structure with modularity *Q* = 0.5112 and weighted mean silhouette *S* = 0.8012. Overall, these statistics suggest that the network can be divided into distinct groups with high intragroup cohesion. Several prominent collaborative groups are identifiable. A prominent group comprises Migliore, Alberto; Conrozier, Thierry; and Chevalier, Xavier, who are prominent and centrally positioned within the network. Their frequent co-authorship and dense local connectivity are consistent with sustained collaboration, which may support multicenter clinical work and the accumulation of more comparable datasets over time.

**Table 4:** Ranking of the top 10 most prolific authors in research concerning IAHA for knee osteoarthritis (2015–2025).

Rank	Author	Count (N)
1	Migliore, Alberto	17
2	Cole, Brian J	16
3	Conrozier, Thierry	15
4	Filardo, Giuseppe	12
5	Chevalier, Xavier	11
6	Kon, Edo	11
7	Hunter, David J	10
8	Zhang, Y	10
9	Di Matteo, Berardo	9
10	Altman, RD	8

IAHA = intra-articular hyaluronic acid.

In addition, authors such as Brian J. Cole appear in relatively central positions linking different parts of the network, suggesting a possible “connector” role that may facilitate cross-cluster exchange.

Overall, the co-authorship structure suggests a field characterized by both stable collaborative cores and a smaller number of cross-cluster links. This topology may support continuity in clinical research programs while still allowing selective exchange across subcommunities, although co-authorship networks alone cannot determine the direction or impact of knowledge transfer.

### 3.5. Analysis of reference co-citation and the intellectual base

Across the 2015 to 2025 IAHA–KOA literature, we extracted 23,610 cited references, which together constitute the dataset’s shared knowledge base. Table 5 lists the top 10 publications ranked by co-citation frequency. Each of these papers was co-cited at least 99 times, and 9 exceeded 100 co-citations, indicating that they are repeatedly used by subsequent studies as common reference points rather than isolated citations.

**Table 5:** Top 10 high-impact co-cited references constituting the intellectual base of research on IAHA for knee osteoarthritis (2015–2025).

Rank	Cited references	Citations
1	Mcalindon TE, 2014, Osteoarthr Cartilage, v22, p363	149^[9]^
2	Patel S, 2013, Am J Sport Med, v41, p356	141^[10]^
3	Kellgren JH, 1957, Ann Rheum Dis, v16, p494	132^[11]^
4	Hochberg MC, 2012, Arthrit Care Res, v64, p465	127^[12]^
5	Bannuru RR, 2019, Osteoarthr Cartilage, v27, p1578	123^[13]^
6	Rutjes AWS, 2012, Ann Intern Med, v157, p180	102^[14]^
7	Bannuru RR, 2015, Ann Intern Med, v162, p46	101^[15]^
8	Cole BJ, 2017, Am J Sport Med, v45, p339	101^[16]^
9	Filardo G, 2015, Am J Sport Med, v43, p1575	101^[17]^
10	Görmeli G, 2017, Knee Surg Sport Tr A, v25, p958	99^[18]^

IAHA = intra-articular hyaluronic acid.

**Figure 9.**
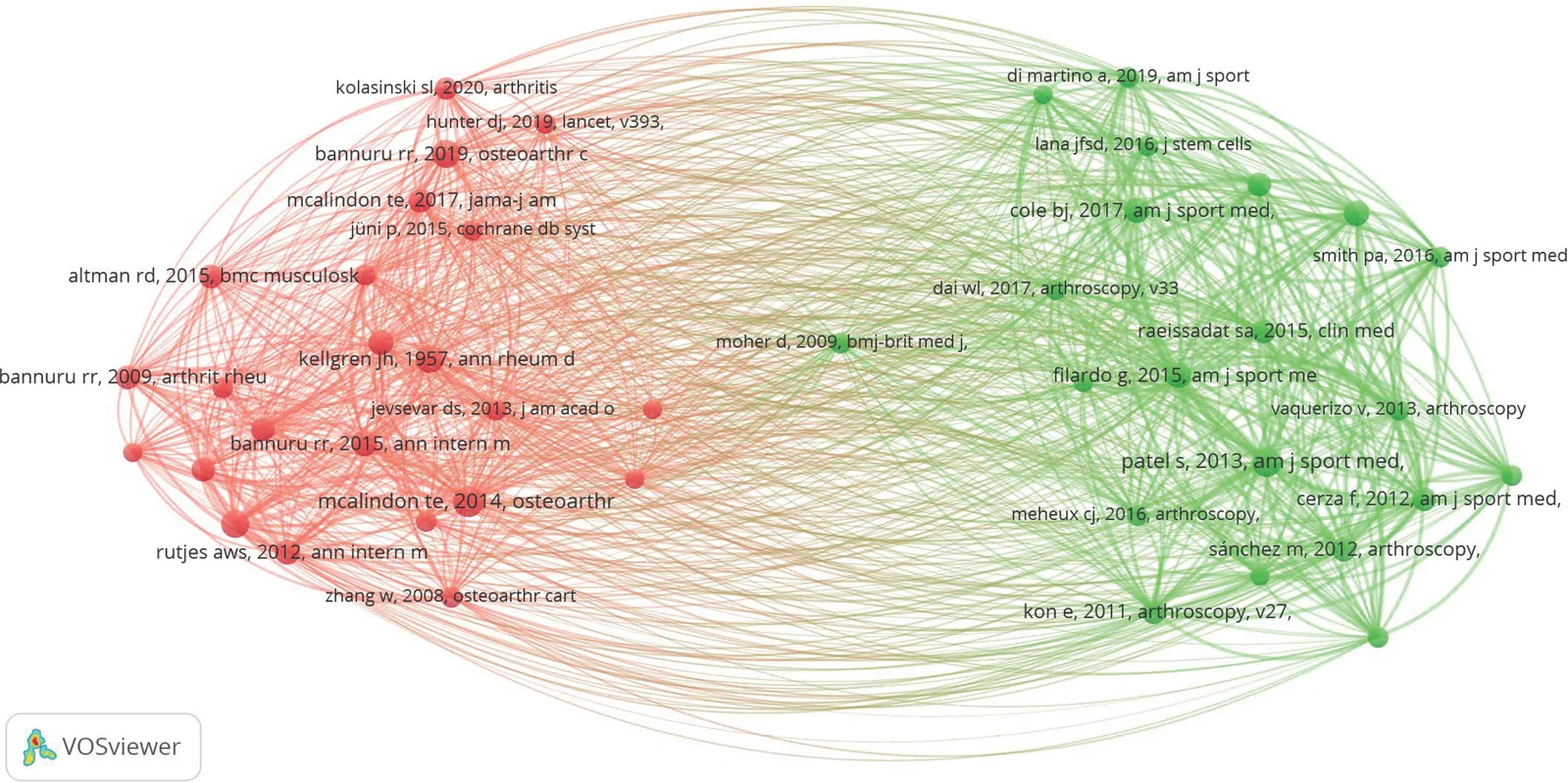
Document co-citation network map elucidating the foundational intellectual base of IAHA research for knee osteoarthritis. IAHA = intra-articular hyaluronic acid.

To visualize how these frequently co-cited works relate to one another, we constructed a document co-citation network in VOSviewer using a minimum threshold of 60 co-citations (Fig. 9). The network highlights several influential nodes that organize much of the field’s shared framing:

Clinical guideline anchors: guideline statements by McAlindon^[9]^ and Bannuru et al,^[13]^ both published in Osteoarthritis and Cartilage, together with guidance associated with the American College of Rheumatology (e.g., Hochberg et al^[12]^), occupy central positions. This centrality indicates the role that these guidelines play in the discussion, comparison, and justification of IAHA, as represented in the literature. Shared diagnostic baseline: the Kellgren–Lawrence grading scale (1957)^[11]^ is represented as a recurrent methodological baseline, primarily due to its continued use in the classification of disease in the context of inclusion criteria in the study of KOA. Evidence synthesis and contention: the meta-analytic research by Rutjes et al^[14]^ and the clinical research by Patel et al^[10]^ are represented as highly connected nodes, indicative of the continued evaluation of the strength, durability, and context-dependent nature of IAHA.

The relatively high interconnectivity of the nodes in the network may indicate the use of a closely linked set of guidelines, classification systems, and syntheses in the construction of arguments and the interpretation of results.

### 3.6. Bibliometric profiling of keywords: co-occurrence, evolution, and density

To characterize major research topics and the relationships among key terms, we conducted a keyword co-occurrence analysis and clustering. Co-occurrence networks help to clarify those terms that co-occur with significant frequency, thus facilitating the process of synthesizing the organization of themes as represented by the existing body of scholarship. By employing VOSviewer, we were able to visualize the organization of keywords; the thickness of the connections between keywords corresponds to the strength of co-occurrence for any given pair of keywords. The minimum threshold for co-occurrence was established at 25 occurrences; this process generated a list of 65 high-frequency keywords for further inclusion. The top 20 keywords by frequency are represented in Table 6. In order to provide different perspectives on the organization of keywords, 3 different standardized VOSviewer views were generated:

**Table 6:** Ranking and distribution of the top 20 high-frequency keywords in research concerning IAHA for knee osteoarthritis.

Rank	Keyword	Frequency	Rank	Keyword	Frequency
1	Hyaluronic acid	642	11	Management	173
2	Knee osteoarthritis	539	12	Knee	162
3	Osteoarthritis	420	13	Hip	148
4	Platelet-rich plasma	342	14	Injection	146
5	Intra-articular injection	309	15	Meta-analysis	118
6	Efficacy	272	16	Mesenchymal stem cells	106
7	Double-blind	253	17	Placebo	106
8	Viscosupplementation	225	18	Therapy	106
9	Pain	214	19	Safety	98
10	Cartilage	204	20	Arthritis	82

IAHA = intra-articular hyaluronic acid.

**Figure 10.**
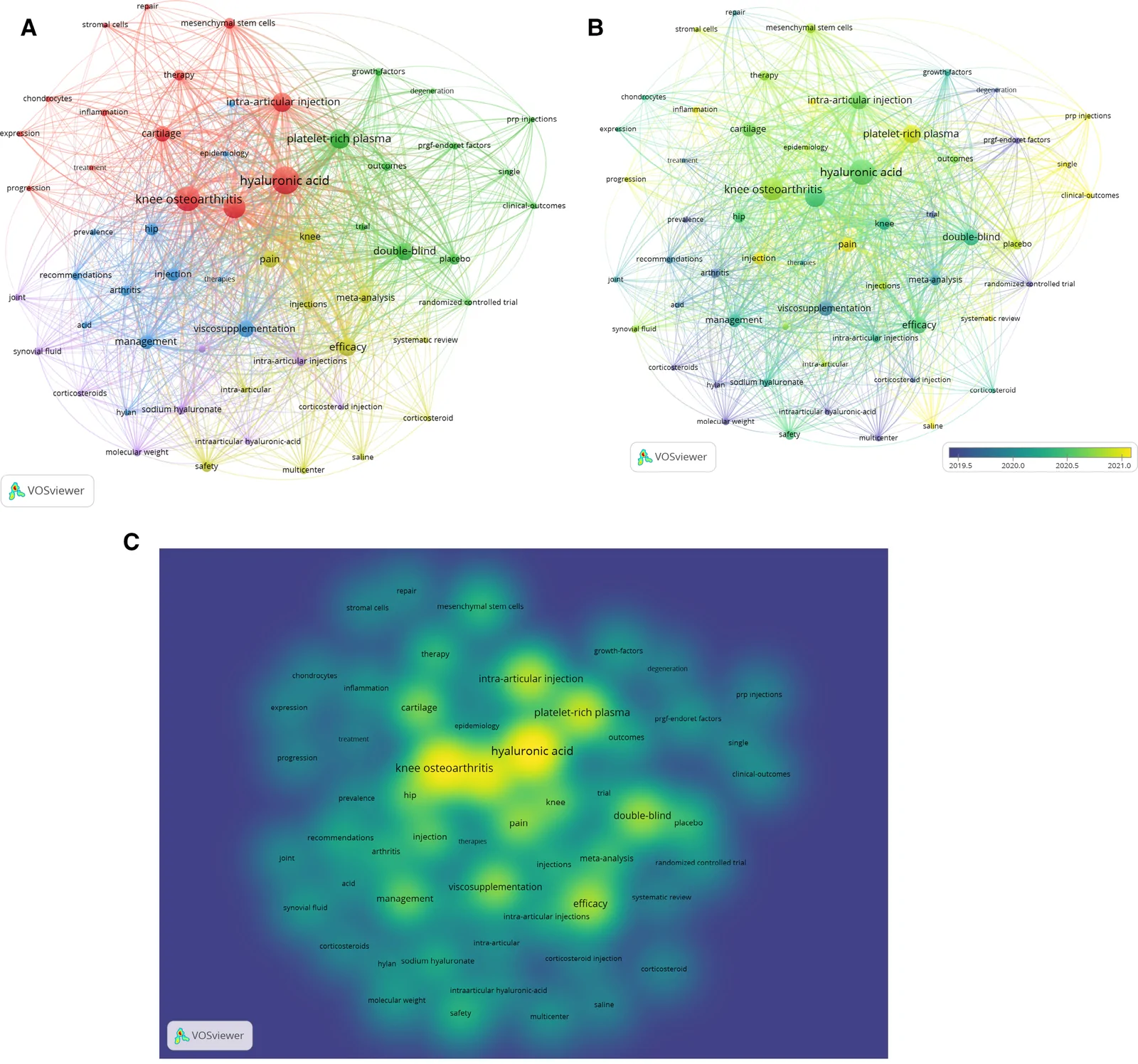
(A) Keyword co-occurrence network map illustrating the thematic landscape of IAHA research. (B) Temporal overlay visualization of keywords illustrating the chronological evolution of research hotspots. (C) Keyword density visualization map highlighting research intensity and hotspot saturation. IAHA = intra-articular hyaluronic acid.

Network visualization (Fig. 10A): provides a visual of the co-occurrence architecture as a whole and the cluster affiliation of keywords.

Overlay visualization (Fig. 10B): provides a visual of the mean year of publication for each keyword.

Density visualization (Fig. 10C): provides a visual of regions of high keyword density, thus indicating regions of high activity with regard to topics.

These visualizations provide a concise overview of the organization of themes within the field of study and highlight the manner in which the focus of the field has evolved over time from previously dominant topics to more contemporary topics of interest.

**Figure 11.**
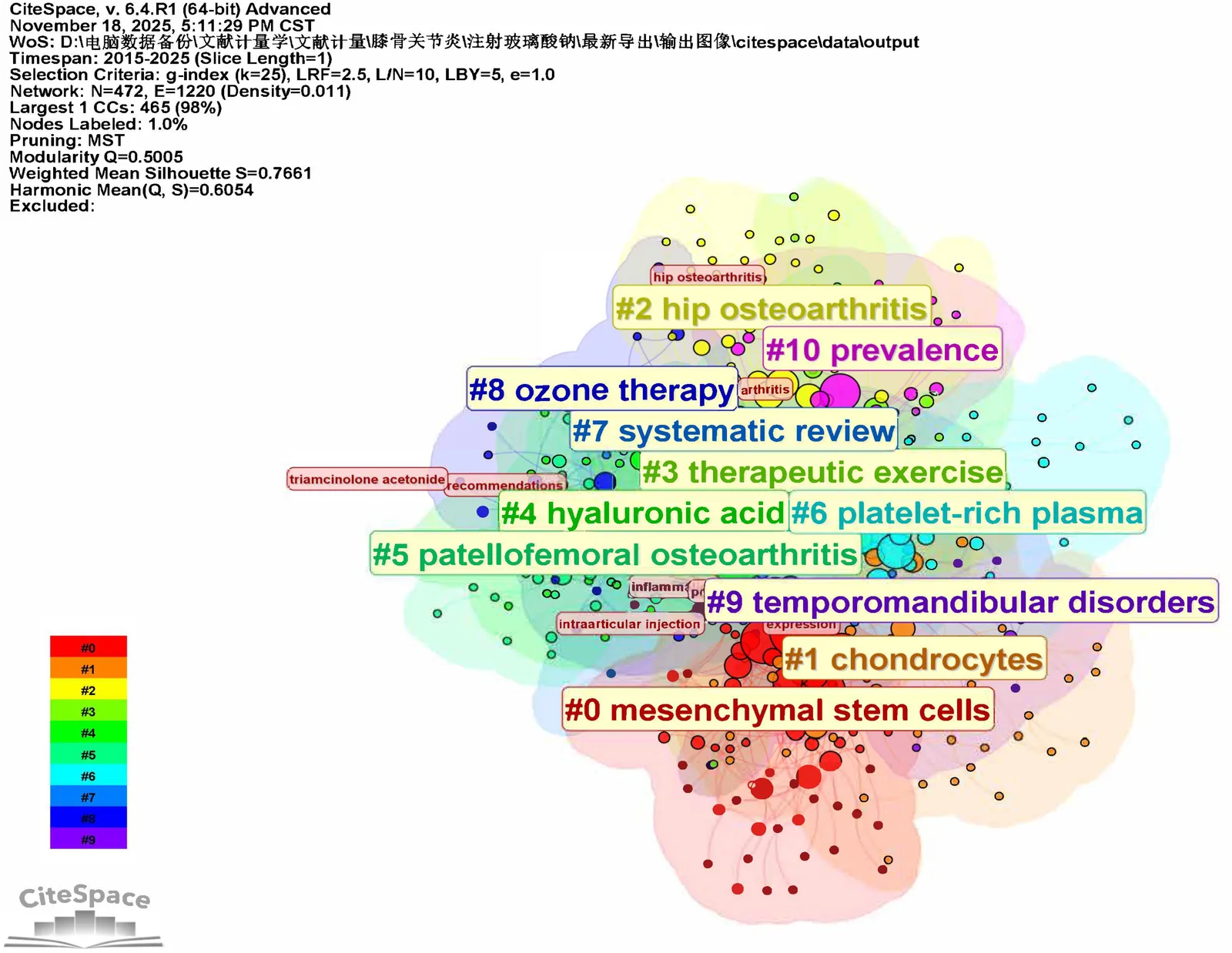
Keyword co-occurrence clustering map illustrating the thematic structure of IAHA research. IAHA = intra-articular hyaluronic acid.

We used CiteSpace to create a keyword clustering map (Fig. 11) to learn more about the subject organization of the field. The network formed 11 main clusters and had a strong structural quality, with a modularity of *Q* = 0.5005 and a weighted mean silhouette of *S* = 0.7661. This means that the clustering solution has a clear community structure, and each cluster is very cohesive.

Clusters were labeled (by CiteSpace) and indexed from #0 to #10: #0 Mesenchymal stem cells (MSCs), #1 Chondrocytes, #2 Hip osteoarthritis, #3 Therapeutic exercise, #4 Hyaluronic acid, #5 Patellofemoral osteoarthritis, #6 Platelet-rich plasma (PRP), #7 Systematic review, #8 Ozone therapy, #9 Temporomandibular disorders, and #10 Prevalence. When considered alongside the VOSviewer density view (Fig. 10C), terms such as “mesenchymal stem cells” and “platelet-rich plasma” appear in high-density regions, consistent with their frequent co-occurrence and strong connectivity within the keyword network.

**Figure 12.**
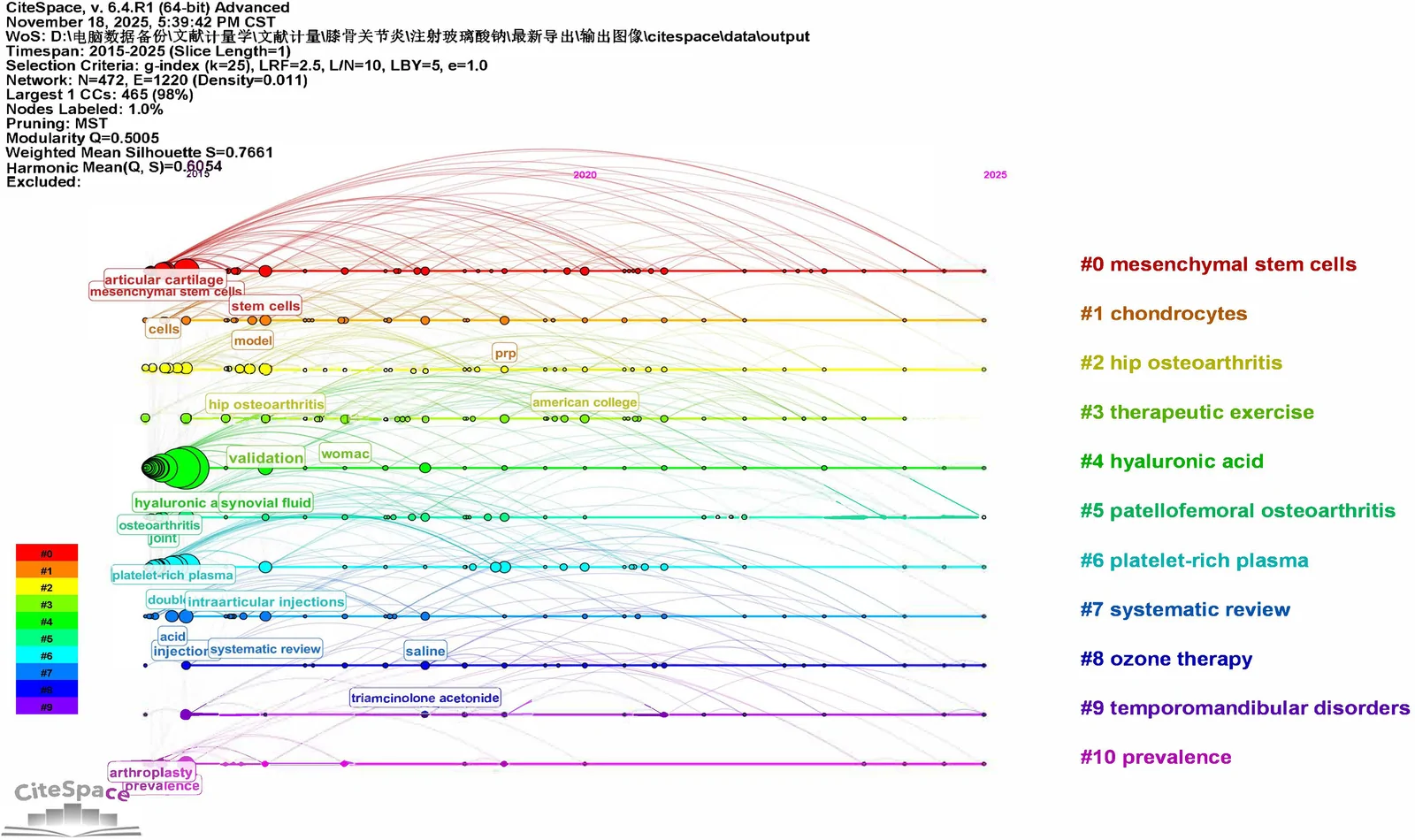
Timeline visualization of keyword clusters illustrating the temporal evolution of research themes.

The keyword timeline (Fig. 12) shows a gradual shift in the reweighting of research focus, rather than a fundamental shift in the thematic foci of previous work, from symptom-oriented interventions to biologic repair concepts. The main cluster #0 MSCs and the PRP cluster #6 indicate a shift in emphasis toward regenerative and orthobiologic interventions. The Chondrocytes cluster provides a mechanistic element, indicating continued interest in cell and extracellular matrix-related phenomena, which are often postulated as a means to achieve a possible effect on the joint structure. Increased anatomical and phenotypic specificity. The development of separate clusters for #2 Hip OA and #5 Patellofemoral OA suggests a trend in some of the literature toward more joint- or compartment-specific research, which could potentially inform more specific phenotypes and, by extension, treatment approaches. Combined and comprehensive management. At the same time, the keyword timeline also shows a continued focus on combined management approaches and the consideration of combination therapy and comprehensive management. The #4 Hyaluronic acid cluster is still a significant theme, and the fact that it often appears alongside biological phrases (like PRP) shows that people are becoming more interested in combination techniques. The fact that #3 Therapeutic Exercise is still visible shows that nondrug management is still a major part of KOA research, and it is commonly discussed alongside injection-based therapy rather than as a competing option.

Extension beyond the knee and the context of the population. The emergence of #9 Temporomandibular diseases presumably signifies the application of IAHA in other synovial joints and the transference of concepts and methodologies beyond KOA-centric research. The #10 Prevalence cluster offers a population-level context, corresponding with an ongoing focus on illness burden and the necessity for scalable management options.

Lastly, even though the timeline patterns show that MSC- and PRP-related topics have become more popular in the last few years, bibliometric trends should be seen as research interest rather than proof of therapeutic advantage. It is expected that future study in these areas will be better if there are clearer standards for protocols (for example, formulation, dose, and delivery schedules), greater links between mechanisms and clinically relevant results, and better quality comparative trials. In parallel, the #7 Systematic review cluster remains important for consolidating rapidly accumulating evidence and informing guideline development as the field evolves.

**Figure 13.**
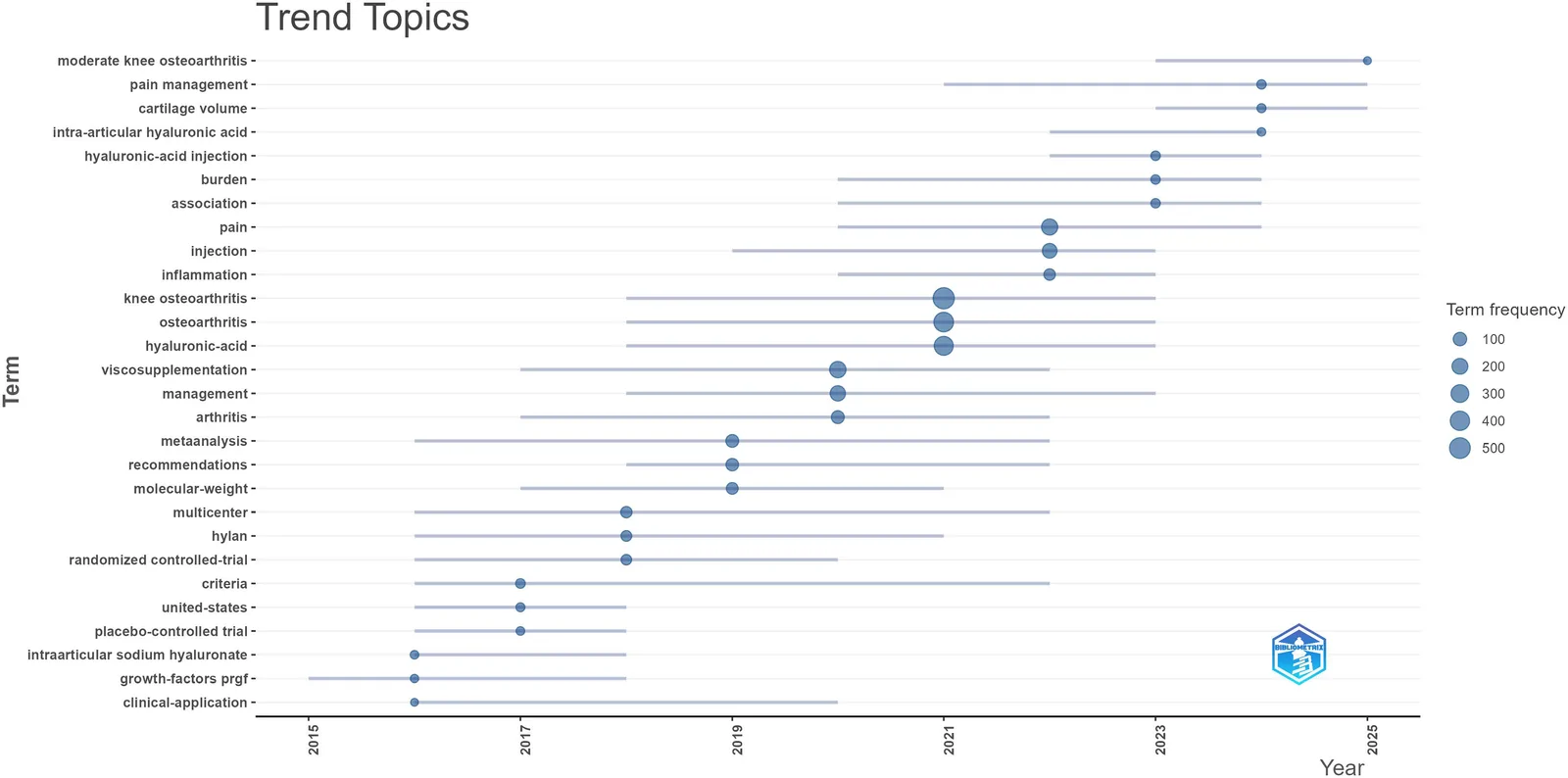
Trend topics analysis of IAHA research for knee osteoarthritis based on the R-bibliometrix package. IAHA = intra-articular hyaluronic acid.

Using the Biblioshiny interface in the bibliometrix R package, we conducted a thematic trend analysis to summarize how topic emphasis changed over time (Fig. 13). The results suggest a progressive shift in the IAHA–KOA literature that can be described in 3 broad phases:

- Mechanistic and product-characterization phase: earlier work more often emphasized core concepts and formulation-related issues, reflected by recurring terms such as “*hyaluronic acid*,” “*viscosupplementation*,” and “*molecular weight*.” This is similar to the emphasis given to the potential relationships between hyaluronic acid properties and lubrication, viscoelasticity, and biological plausibility.
- Clinical evaluation phase: in subsequent years, increased emphasis was given to terms such as “randomized controlled trial,” “placebo-controlled,” and “multicenter,” indicating a period where efficacy and safety were regularly evaluated in a comparative manner.
- Synthesis and guidance phase: more recent trends are dominated by evidence-integration terms such as “*meta-analysis*,” “*recommendations*,” and “*pain management*,” suggesting that the field has moved toward consolidating heterogeneous findings and translating them into guidance for clinical decision-making.

Overall, the trend-topic outputs indicate that IAHA research for KOA has shifted from formulation/mechanism-focused discussions toward comparative clinical evaluation and, more recently, toward evidence synthesis and practice-oriented recommendations. Notably, these bibliometric patterns capture changes in research attention rather than definitive clinical effectiveness; nonetheless, they provide a useful context for interpreting how the evidence base has developed and why conclusions may still differ across guidelines.

**Figure 14.**
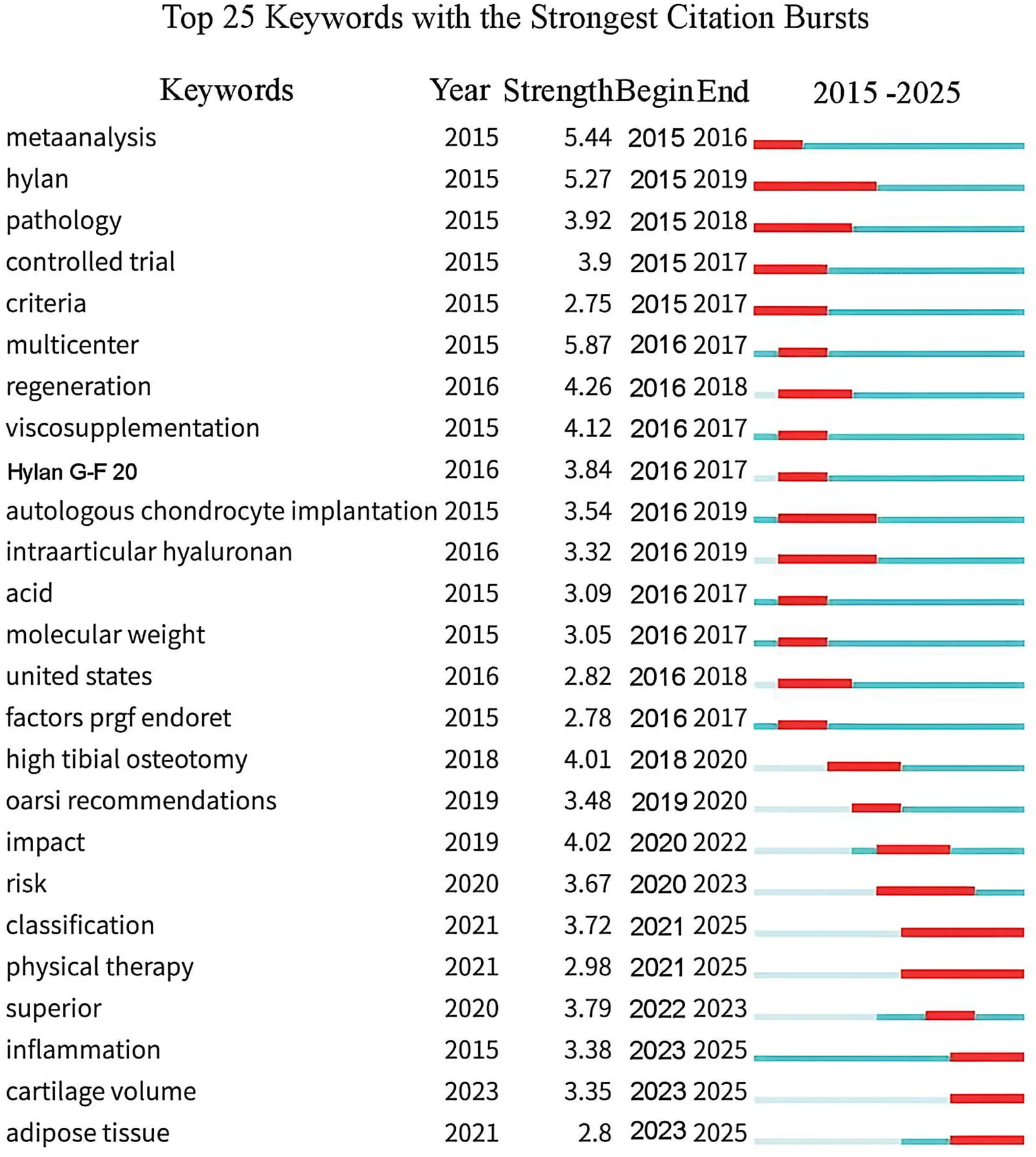
Top 25 keywords with the strongest citation bursts in research on IAHA for knee osteoarthritis. IAHA = intra-articular hyaluronic acid.

The keyword burst analysis (Fig. 14) offers a time-sensitive view of topics that received unusually rapid increases in attention. Overall, the bursts point to 2 major changes in focus during the course of the study.

There was more of a focus on clinical positioning and risk assessment between 2018 and 2020.

From 2018 to 2020, burst terms demonstrate that more attention was paid to how IAHA is used in practice and how it is judged in a way that is more aware of policy and safety. The rise of “OARSI recommendations” (2019–2020) coincides with the growing interest in making decisions based on guidelines. The concurrent increase in both “impact” and “risk” suggests a shift in the discourse from a focus on short-term effectiveness to a focus on benefit-risk assessment, patient selection, and pragmatic considerations of administration. The appearance of “regeneration” at this time suggests that the discussion was expanding beyond viscoelastic supplementation, at least, to consider the possibility of effects on biology or the cellular microenvironment. The fleeting nature of the peak in scholarly activity, however, suggests that investigators were more interested in the topic than in exploring the underlying mechanisms.

Since 2021, more attention has been paid to biologics, structural results, and multimodal treatment.

Beginning in 2021, bursts will increasingly use terms associated with orthobiologics and objective result evaluation. The rise in interest in “adipose tissue” from 2023 to 2025 suggests that individuals are growing more interested in products made from adipose tissue and how they could be employed with IAHA, such as in certain research as a carrier or scaffold concept. The significance of “cartilage volume” indicates an emphasis on imaging-based or structural outcomes, along with initiatives to examine changes beyond symptom scores. In the meantime, the ongoing “physical therapy” (2021–2025) indicates that individuals are still interested in rehabilitation and combination management measures. This indicates that IAHA is frequently discussed within the context of broader multimodal care pathways rather than as an independent treatment.

## 4. Discussion

### 4.1. Global research topography and collaborative architecture

To the best of our knowledge, this study provides a comprehensive bibliometric overview of the global research landscape of IAHA for KOA over the past decade. The steady increase in publication output may reflect sustained academic and clinical interest in minimally invasive and joint-preserving treatment strategies for KOA, particularly in aging populations.

Collaboration network analyses demonstrated that several high-output institutions, including Rush University, the University of Washington, and the University of Toronto, occupied central positions within the research network and formed recognizable collaborative clusters. However, cross-regional and transcontinental cooperation appeared relatively limited, suggesting that research activity remains concentrated within regional collaborative networks.

This pattern may partially reflect differences in research priorities, clinical practice patterns, and guideline recommendations across countries and institutions. Strengthening international and multicenter collaboration may improve data comparability and promote more standardized approaches in future IAHA-related research.

### 4.2. Scholarly visibility and dissemination dynamics

Journal-level analysis showed that most publications in this field were published in specialty journals within orthopedics, sports medicine, rheumatology, and rehabilitation. These journals were primarily categorized within JCR/CAS Q1 to Q3 classifications in their respective research areas, reflecting the specialized clinical and translational focus of IAHA-related research in KOA management.

The current literature has mainly focused on clinical efficacy, safety evaluation, and comparative therapeutic strategies, whereas relatively fewer studies have explored mechanistic pathways and translational aspects. This publication pattern may partially explain why the literature is concentrated within specialty journals rather than broader general medical journals.

In recent years, increasing research attention has been directed toward precision medicine approaches and phenotype-based treatment strategies for KOA. Future studies may further explore patient stratification, multimodal therapeutic approaches, and the biological interactions between IAHA and the joint microenvironment to better understand IAHA-related therapeutic research in KOA.

### 4.3. Thematic evolution and emerging research trends

Keyword burst and co-occurrence analyses demonstrated evolving research interests within the IAHA-related literature for KOA. Earlier studies mainly focused on viscosupplementation, pain relief, and functional improvement, whereas more recent studies have increasingly emphasized regenerative medicine-related topics and combination-based therapeutic strategies.

In particular, keywords related to MSCs, PRP, and orthobiologic therapies have shown increased research attention in recent years. These findings suggest that current research is expanding beyond conventional symptomatic management toward exploring multimodal and biologically oriented treatment approaches. Comparative evidence from network meta-analyses has contributed to ongoing discussions regarding the relative effectiveness of IAHA and other therapeutic interventions for KOA.^[15]^ In addition, several randomized controlled trials comparing IAHA with PRP have reported heterogeneous findings, including studies by Cole et al,^[16]^ Filardo et al,^[17]^ and Görmeli et al.^[18]^ These influential studies have helped shape the current research focus on orthobiologics and combination-based treatment strategies.

In addition, the increased occurrence of epidemiology-related and patient stratification-related keywords may reflect growing academic interest in individualized treatment strategies and risk-based management approaches for KOA. The present bibliometric analysis summarizes the evolving research landscape of IAHA for KOA and may provide useful references for future clinical and translational studies in this field.

### 4.4. Methodological limitations and reflexive critique

We followed a transparent and systematic approach, although with some limitations. First, our analysis was mainly based on the WoSCC. Even though this database has the advantage of providing consistent and high-quality information, it may have omitted some important studies that are not indexed in this database, such as those that are included in Scopus or regional databases like CNKI. Second, only studies published in English may have been considered, although this field has seen significant contributions from researchers in other regions, especially China, where this field has been particularly active. Third, there are some issues of “time-lag effects,” as important studies published over the last approximately 24 months may not have achieved enough citations to be considered as “bursts,” although they are expected to have an important impact on the field in the near future.

## 5. Conclusion

This bibliometric study provides a comprehensive overview of global research trends and hotspots related to IAHA for KOA from 2015 to 2025. Publication output increased steadily over the past decade, with China and the United States contributing the largest number of publications. Collaboration network analysis indicated that several regional research clusters have been established, although international and transcontinental cooperation remains relatively limited.

Keyword co-occurrence, burst detection, and thematic analyses suggested that research attention has gradually expanded from conventional viscosupplementation and symptom-oriented outcomes toward regenerative medicine-related topics, including PRP, MSCs, and combination-based therapeutic approaches. These findings should be interpreted as changes in academic focus and publication trends rather than evidence of established clinical superiority or guideline-level consensus.

Future IAHA-related research may benefit from more standardized study designs, harmonized outcome measures, multicenter collaboration, and further mechanistic investigations. Such efforts may improve the comparability and interpretability of evidence in this field and contribute to a better understanding of IAHA-related research directions in KOA management.

## Author contributions

**Conceptualization:** Zhijun Yang, Youkang Dong.

**Data curation:** Zhijun Yang, Penghui Li, Yu Xu.

**Formal analysis:** Zhijun Yang, Penghui Li, Yu Xu.

**Investigation:** Zhijun Yang, Penghui Li, Yu Xu.

**Methodology:** Feifei Shen, Jie Li.

**Validation:** Feifei Shen, Jie Li.

**Funding acquisition:** Youkang Dong.

**Project administration:** Youkang Dong.

**Supervision:** Youkang Dong.

**Writing – original draft:** Zhijun Yang.

**Writing – review & editing:** Youkang Dong.


