# Supplementary material for: Global trends and hotspots of intra-articular hyaluronic acid therapy for knee osteoarthritis: A bibliometric study
Source: Medicine (Baltimore). 2026 Sep 11;105(37):e50660. doi: 10.1097/MD.0000000000050660 (PMC13574390; doi:10.1097/MD.0000000000050660)
Supplement: Supplementary file 1 [file medi-105-e50660-s001.docx]

Supplementary Table S1. Keyword normalization dictionary

| Original term | Unified term |
| --- | --- |
| hyaluronic-acid | hyaluronic acid |
| intraarticular injection | intra-articular injection |
| intraarticular injections | intra-articular injections |
| platelet rich plasma | platelet-rich plasma |
| PRP | platelet-rich plasma |
| mesenchymal stem-cells | mesenchymal stem cells |
| metaanalysis | meta-analysis |
| randomized controlled-trial | randomized controlled trial |
| molecular-weight | molecular weight |
| articular-cartilage | cartilage |
| synovial-fluid | synovial fluid |
| factors prgf-endoret | PRGF-Endoret factors |

Note: Only keywords requiring normalization are listed in this table. Keywords already consistent with the unified terminology were retained without modification.
